# Application of Arsenic to Teeth

**Published:** 1859-10

**Authors:** 


*Application of Arsenic to Teeth.*—It is stated in the New York Herald, that Lieut. Stanford, late of the United States revenue cutter, Harriet Lane, was supposed to have lost his life from the effects of arsenic applied to a tooth, for the purpose of destroying the nerve.

If this be true, four times as much arsenic must have been used as is required to destroy the vitality of the pulp of a tooth, and it must have been applied very carelessly. In the use of this agent medicinally, the tenth part of a grain is often administered at a time, and the fortieth or fiftieth of a grain is sufficient to destroy the nerve of a tooth. After being placed upon the exposed nerve, the cavity in the tooth should be securely filled with softened wax, to prevent the possibility of the escape of the agent into the mouth.

The manner in which we have it prepared for use is, to mix gr. 1 of arsenious acid with the same quantity of sulph. morphia. The two are thoroughly incorporated and then divided into thirty parts. Each of these powders is put into a paper by itself and put by for use. In applying it to a tooth, a small dossil of raw cotton is moistened in creosote, and then placed on the arsenical powder, which it readily absorbs. This done, the cotton is placed carefully over the exposed pulp, and the cavity sealed in the manner as stated above. It is permitted to remain in the tooth from seven to ten hours; it is then removed, and the pulp completely extirpated to the extremity of the root.

In removing the powder about one-tenth of it remains adhering to the paper, so that not more than the fortieth part of a grain is actually applied to the tooth. This quantity is as effective in the destruction of the vitality of the pulp of a tooth as half a grain would be.

